# Probing the missing mature β-cell proteomic landscape in differentiating patient iPSC-derived cells

**DOI:** 10.1038/s41598-017-04979-w

**Published:** 2017-07-06

**Authors:** Heidrun Vethe, Yngvild Bjørlykke, Luiza M. Ghila, Joao A. Paulo, Hanne Scholz, Steven P. Gygi, Simona Chera, Helge Ræder

**Affiliations:** 1 KG Jebsen Center for Diabetes Research, Department of Clinical Science, University of Bergen, Bergen, Norway; 2 Department of Cell Biology, Harvard Medical School, Boston, MA USA; 3 Department of Transplant Medicine, Oslo University Hospital, Oslo, Norway; 4 Department of Pediatrics, Haukeland University Hospital, Bergen, Norway

## Abstract

MODY1 is a maturity-onset monogenic diabetes, caused by heterozygous mutations of the *HNF4A* gene. To date the cellular and molecular mechanisms leading to disease onset remain largely unknown. In this study, we demonstrate that insulin-positive cells can be generated *in vitro* from human induced pluripotent stem cells (hiPSCs) derived from patients carrying a non-sense *HNF4A* mutation, proving for the first time, that a human *HNF4A* mutation is neither blocking the expression of the insulin genes nor the development of insulin-producing cells *in vitro*. However, regardless of the mutation or diabetes status, these insulin-producing cells are immature, a common downfall off most current β-cell differentiation protocols. To further address the immature state of the cells, *in vitro* differentiated cells and adult human islets were compared by global proteomic analysis. We report the predicted upstream regulators and signalling pathways characterizing the proteome landscape of each entity. Subsequently, we focused on the molecular components absent or misregulated in the *in vitro* differentiated cells, to probe the components involved in the deficient *in vitro* maturation towards fully functional β-cells. This analysis identified the modulation of key developmental signalling pathways representing potential targets for improving the efficiency of the current differentiation protocols.

## Introduction

Monogenic disorders are caused by germline single gene defects where different mutations in the causal gene usually trigger a defined disorder with characteristic clinical features. The identification of the genes and molecular networks underlining monogenic disorders allows for unbiased characterization of the basic mechanisms regulating cell-fate decisions during development and disease onset. This approach also facilitates the understanding of the aetiology of the more prevalent corresponding multifactorial diseases as well as general developmental aspects. One such example is Parkinson disease, where the study of its few monogenic variants tremendously boosted the knowledge of the mechanisms involved in neuronal differentiation, homeostasis and disease initiation^1^. Similarly, MODY (Maturity Onset Diabetes of the Young) represent a distinct group of diabetic disorders characterized by the impairment of pancreatic β-cells (the insulin-producing cells) caused by an autosomal dominantly inherited mutations. Due to their unambiguous and inheritable genetic readout, MODYs are ideal tools for elucidating the molecular and cellular basis involved in β-cell differentiation and failure.

Studies on human patients are extremely challenging and have inherent technical and ethical limitations. As a result, most research on human diseases is based on model organisms and *in vitro* approaches. Moreover, as many of the currently available murine models of MODY fail to accurately replicate the equivalent human conditions^2–5^, the efforts for understanding the dynamic of β-cell failure focuses mostly on *in vitro* setups. Consequently, the past decade has seen the development of several *in vitro* directed differentiation protocols using human pluripotent stem cells (hiPSCs) as a renewable resource for making insulin-producing cells as models for diabetes^6–10^.

The protocols reported in 2014 by *Rezania et al*. and *Pagliuca et al*. shared a similar stepwise directed differentiation strategy attempting to mimic different stages of the embryonic development of β-cells by modulating similar developmental signalling pathways. Both protocols achieved to generate β-like cells on a comparable timescale, employing slightly different compounds and working concentrations. A novel protocol reported by *Russ et al*. in 2015 suggested simplifications of the protocols without compromising differentiation efficacy, i.e. by omitting BMP inhibitors at the pancreas specification stage and by finding that retinoic acid alone was sufficient to generate more than 98% of PDX1+ pancreatic progenitors^8^. To date and to our knowledge there have been no reports of the use of these protocols in the differentiation of hiPSCs from patients with monogenic diabetes (MODY or permanent neonatal diabetes of the young, PNDM). With regards to MODY, differentiation of hiPSCs was recently reported using different protocols: from MODY5 patients towards pancreatic progenitor cells^11^, and from MODY2 patients towards β-like cells^12^.

However, as the current knowledge of β-cell differentiation process during development is incomplete, a common problem of the available *in vitro* differentiation protocols is the production of mostly immature “β-like cells”^13^ unable to perform accurate glucose-stimulated insulin secretion unless they are transplanted into mice and allowed to mature *in vivo* ^14^. This consistent failure of obtaining mature functional insulin-producing cells *in vitro* signifies the absence of a maturing/differentiating factor or factors present *in vivo*, however absent in the current protocols. Correspondingly, finding and characterizing this developmentally important molecular network required for the final maturation of β-cell *in vitro* is urgently needed in order to generate functional insulin-producing cells. Most current attempts towards characterizing β-cell molecular networks are based on next generation sequencing tools such as RNA-seq. Despite the undeniable power and sensitivity of the transcriptomics methods the progress is slow, hence there is also a need for complementary characterizing methods, such as proteomics methods. An increasing number of studies have reported consistent and biologically relevant differences when comparing transcriptomics and proteomics data^15, 16^. These discrepancies are explained by the different dynamics of the RNA and protein products. As an example the ribosome may alter the translational efficiency of mRNA at the initiation and elongation stages^17^. Furthermore, many cellular signals do not activate the transcription of the relevant downstream pathway components, as these proteins have already been synthetized in the cells and are regulated by post-translational modification, such as in the case of insulin signalling. Moreover, the half-lives of transcripts and their respective protein products are different, i.e. with situations where the protein is persistently involved in cellular processes after the disappearance of the corresponding transcript. In any of these cases, transcriptomics tools will fail to detect correctly the changes in gene product abundance or signalling patterns.

Here we used a combination of global proteomics and cellular biology techniques to investigate the differentiation capacity of insulin-producing cells using a seven-step differentiation protocol (as established by *Rezania et al*.), generated from either healthy subjects or MODY1 (i.e. *HNF4A gene* mutation carrying) patients. Next, we compared the *in vitro* stage 7 (S7) cell proteome with human pancreatic islet proteome and identified differentially expressed proteins as well as specific molecular networks distinguishing the end-stage *in vitro* S7 cells from the bona-fide islet cells.

## Results

### *HNF4A* mutation (MODY1) does not prevent the formation of insulin^+^ cells *in vitro*

**Figure 1:**
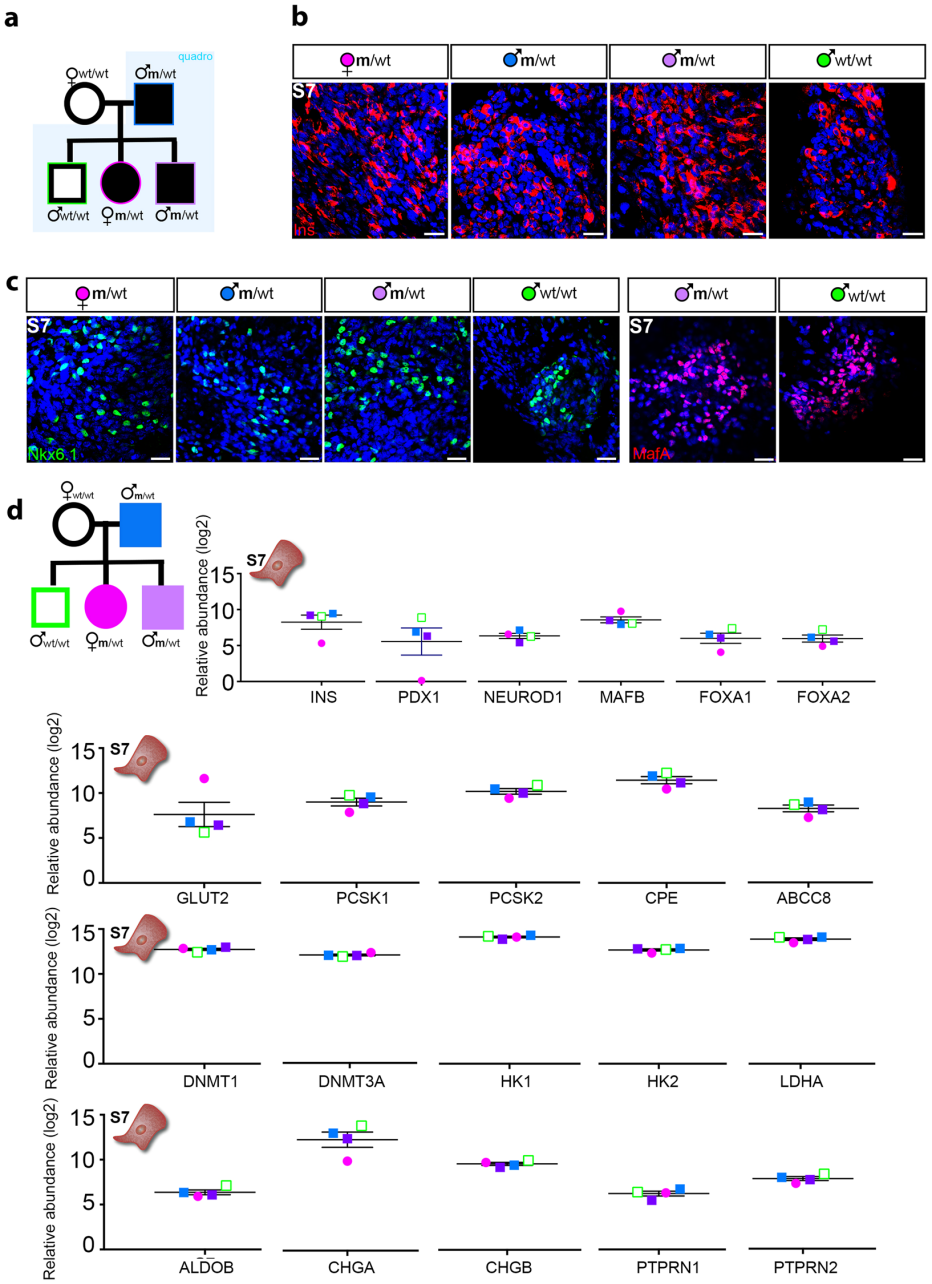
Generation of patient-specific insulin-producing cells from a MODY1 quadro. (**a**) Patient-specific iPSCs were used from four family members (quadro) of a MODY1 family, including a parental diabetic mutation carrier (shown as blue filled square), and three offspring; one diabetic mutation carrier (purple filled square), one non-diabetic mutation carrier (pink filled circle), and non-diabetic non-mutation carrier (green unfilled square). (**b**) Differentiation of the four hiPSC lines towards insulin-producing cells were repeated three times (n = 3) following the seven-stage protocol (as established by Rezania *et al*.), and resulted in insulin+ cells (red) from all four hiPSC lines regardless of mutation status. (**c**) The S7 cell population also expressed *NKX6.1* (turquoise) and *MAFA* (magenta). (**d**) Quantitative proteomics detected insulin along with other β-cell specific markers in all subjects regardless of mutation status. Each patient specific data point is shown in the colours as described in a). Data are shown as mean with SEM. The y-axis shows the relative abundance of each protein (log2) compared to all the ten samples of a TMT ten-plex as further explained in Methods. Scale bar: 25 µm.

We first inquired if the *HNF4A* mutation or diabetes status prevented the differentiation of insulin^+^ cells *in vitro*. As the limited accessibility to human samples and the increased number of variables inherent to any *in vitro* differentiation protocol rule out a differential quantitative analysis, we focused on whether insulin^+^ cells are present or, alternatively absent in each sample (qualitative assessment). To answer this question, skin fibroblasts from a healthy family member and mutation carriers before and after the onset of diabetes from a MODY1 cohort quadro (n = 4, see Fig. 1a) were reprogrammed. The “MODY1 cohort quadro” refers here to the four family members, including a parental diabetic mutation carrier, and three offspring: one diabetic mutation carrier, one non-diabetic mutation carrier, and non-diabetic non-mutation carrier (family control). The synchronous differentiation of the four hiPSCs lines towards insulin-producing cells (n = 3 repeated rounds of differentiation) was done following the seven step differentiation protocol established by Rezania *et al*. (Fig. S1) and resulted in the successful generation of insulin^+^ cells in all four samples regardless of the mutation or diabetes status (Fig. 1b). The Stage7 cell population (S7 thereafter) also expressed key markers of pancreatic β-cells, including NKX6.1 and MAFA (Fig. 1c). Furthermore, the global proteomic comparison of the final stage (S7) cells derived from MODY1-mutation carriers and their family control detected in all samples similar levels of insulin and most key β-cell specific markers, regardless of the mutation or diabetes status (Fig. 1d), confirming the initial immunofluorescence (IF) observations. Overall, these data suggest that the *HNF4A* mutation is neither blocking the expression of the insulin genes nor the development of insulin-producing cells *in vitro*.

### Differentiated S7 cells are characterized by high heterogeneity regardless of the *HNF4a* mutation and disease status

**Figure 2:**
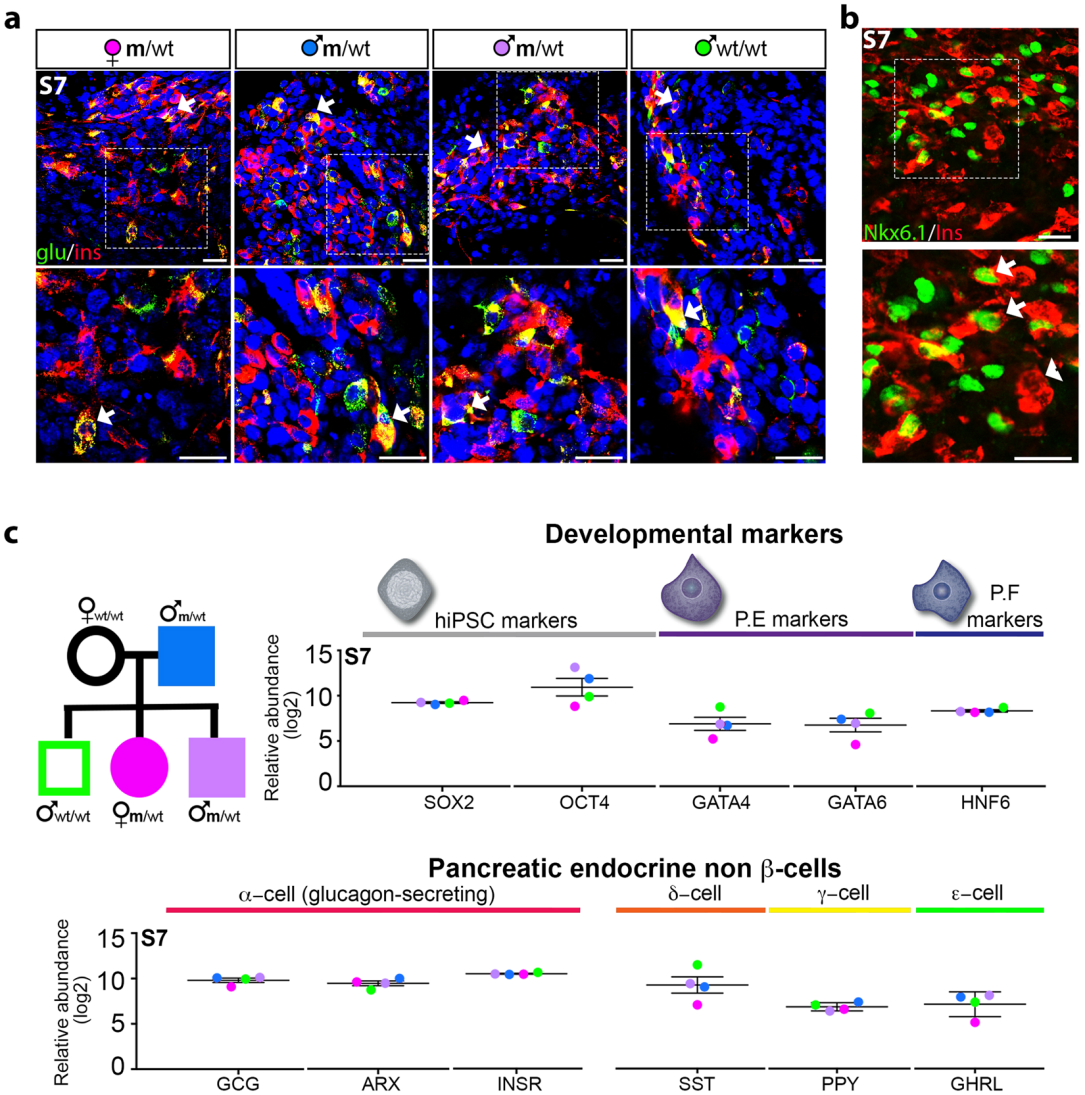
Cellular heterogeneity. (**a**) Co-expression of glucagon (green) and insulin (red) in S7 cells (arrows). (**b**) Insulin+ S7 cells (red) positive (arrows) or negative (arrowhead) for NKX6.1 transcription factor (green). (**c**) Relative abundance (log2) of non-β endocrine islet cells markers in S7 cells compared to all the ten samples of a TMT 10-plex as further explained in Methods. Scale bar: 25 µm.

As reported previously^9^, we observed the co-expression of other islet hormones in the S7 cell population in all samples analysed regardless of mutation status. The co-expression was observed by IF either in the same cell (bi-, multi-hormonal cells) or in different cells belonging to the same S7 pool (Fig. 2a). Moreover, in all S7 insulin^+^ cell populations we observed heterogeneity, with some insulin^+^ cells co-expressing the β-cell marker NKX6.1 (Fig. 2b, arrows), while others did not (Fig. 2b, arrowhead).

The global proteomic comparison of the MODY-derived S7 samples and their family control confirmed these results, with significant levels of non-β endocrine islet cells markers, progenitor cell markers as well as stem cell markers being detected in all samples (Fig. 2c). In our S7 cell populations we detected specific markers of pancreatic δ-cells (somatostatin (SST)), pancreatic α-cells (glucagon (GCG), the α-cell specific transcription factor ARX, insulin receptor (IR) and proconvertase 2 (PCSK2)), pancreatic γ-cells (pancreatic polypeptide (PPY)) and ε-cells (ghrelin (GHR)).

These data suggest that neither HNF4A mutation nor the diabetic status impose a regenerative-driven demand promoting an exclusive β-cell fate. However, the limited number of samples included in this study does not qualify for a reliable quantitative marker analysis between the different S7 samples, hence we cannot exclude that HNF4A mutation or the diabetes status might moderately promote a certain cell fate.

### *HNF4A* mutation does not interfere with β-cell maturity markers acquisition *in vitro*

**Figure 3:**
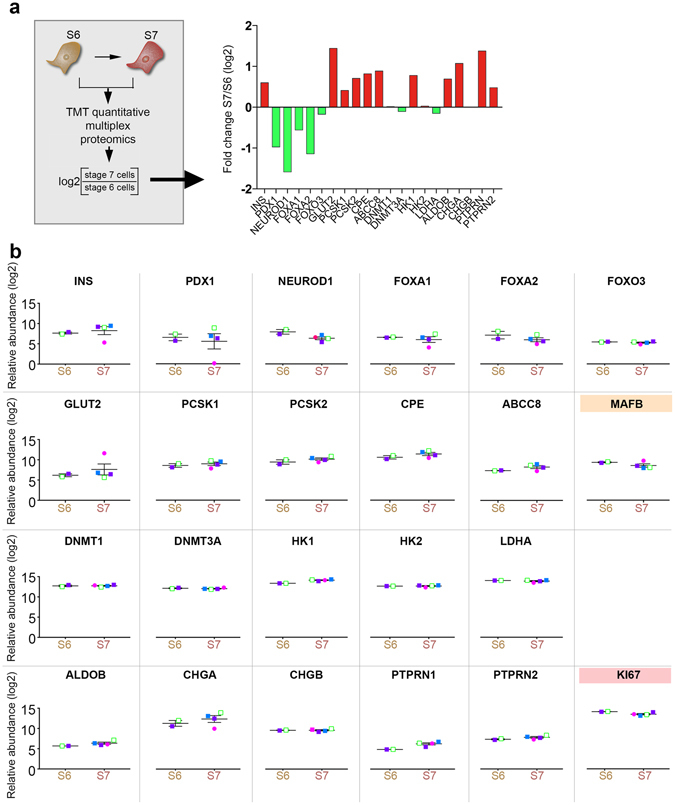
Proteomics comparison of S6 and S7 cells. The relative abundance of proteins important for β-cell function are represented between two S6 samples (one mutation carrier and one family control) and four S7 samples (three mutation carriers and one family control without the mutation). Each patient specific data point is shown in the colours as described in 1a). Due to the limited number of samples allowed in one TMT 10-plex (n = 10), data from two S6 samples are shown. a) Fold difference (log2) between S6 and S7 (**b**) Relative abundance (log2) between S6 and S7 cells. All plots show mean protein level and standard error of mean (SEM).

We then investigated if the *HNF4A* mutation or diabetes status impeded the initial *in vitro* maturation steps of the differentiated insulin^+^ cells. We addressed this question by performing global proteomics comparison of Stage 6 cell population (S6 thereafter) and S7 cells derived from MODY1 mutation carriers and their family control without the mutation. The comparison revealed that, in all the samples analysed and regardless of mutation or diabetes status, several but not all β-cell specific markers including those involved in glucose sensing, regulation of glucose stimulated insulin secretion (thereafter GSIS) and insulin biosynthesis, showed an increasing trend at S7 compared to the previous stage (S6) (Fig. 3a and b). The increasing trend for insulin protein abundance was also in accordance with the IF staining data for insulin (Supplementary Fig. 1). Moreover, the levels of MAFB transcription factor decreased between S6 and S7 cells, consistent with previous studies reporting the switch from MAFB to MAFA expression during β-cell maturation^18^ (Fig. 3b, orange background).

### Despite gaining certain maturity markers, the S7 insulin+ cells are functionally immature *in vitro*, regardless of the *HNF4a* mutation and diabetic status

**Figure 4:**
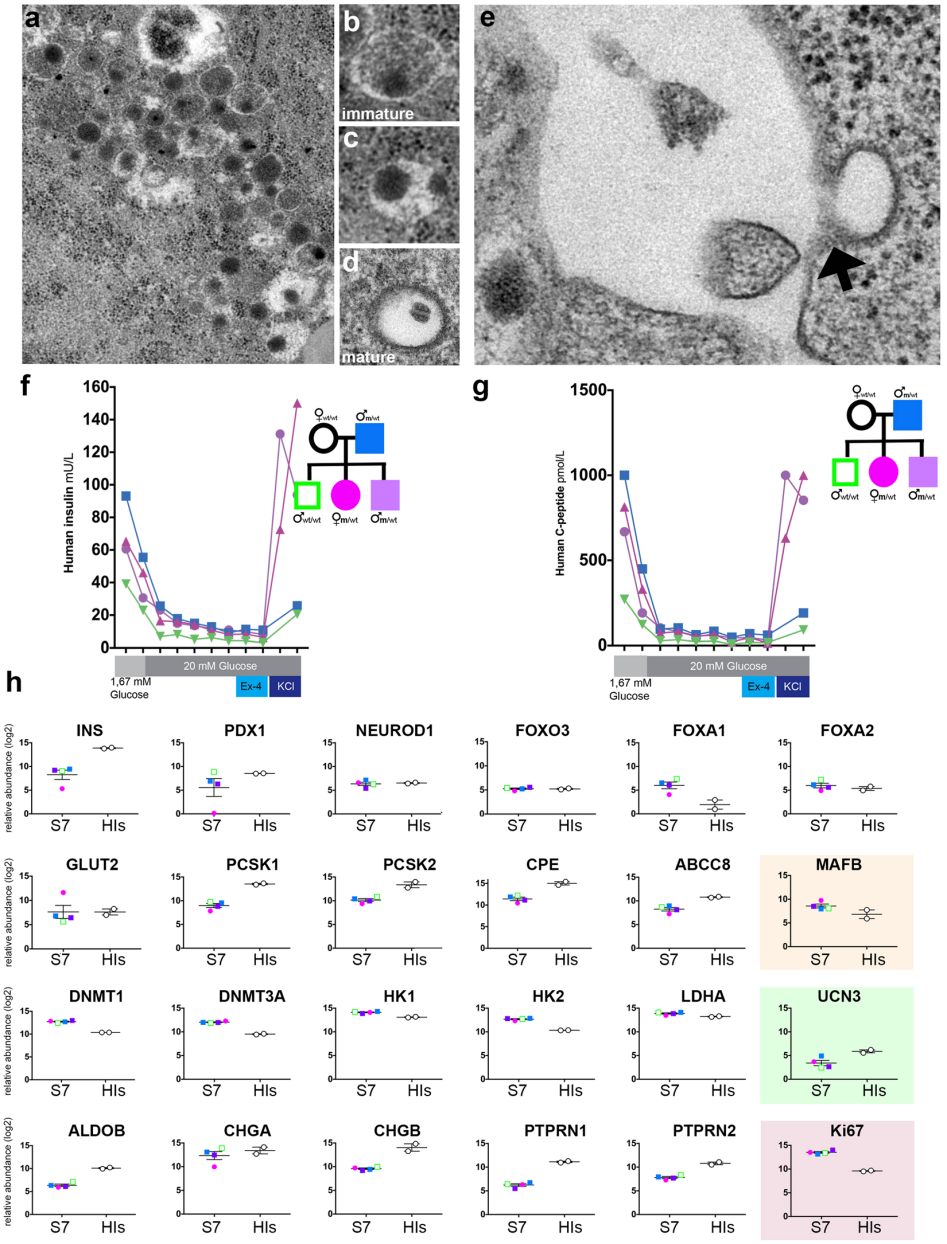
(**a**) Representative transmission electron microscopy images showing fields of endocrine granules in S7 cells. The S7 cells subjected to TEM analysis were not glucose-stimulated. Scale: 25 k magnification. Insulin granules in human β-cells are typically categorized into three main types: (**b**) pale, diffuse grey core (immature granule), (**c**) dense round core without marked edge, and (**d**) dense round core with marked edge (mature granule) Scale: 25k magnification for (**b**), (**c** and **d**). (**e**) Exocytosis of endocrine granule (black arrow). Scale: 80k magnification. **f)** Human Insulin secretion (mU/L) of S7 cells following GSIS. (**g**) Human C-peptide secretion (pmol/L) of S7 cells following GSIS. (**h**) Proteomics comparison of β-cell markers in S7 cells (each patient specific data point is shown in the colours as described in 1a), and human islets (HIs) (shown as white filled circles). All plots show mean protein level with SEM.

To investigate if the maturation status of the S7 insulin^+^ cells is similar to the mature bona-fide β-cells we first performed ultrastructural analysis by transmission electron microscopy (TEM) and identified fields of endocrine granules with different core densities in S7 samples (Fig. 4a–d), consistent with the three types of insulin granules described in mature human β-cells^9^. There were no differences between endocrine granules from mutation-carriers and healthy family control; therefore representative pictures are shown for the healthy control (Fig. 4a–d). These results suggest that despite the S7 cells containing a heterogeneous population of insulin^+^ cells (Fig. 4b and d, respectively), at least some of these insulin^+^ cells are capable of insulin processing and secretion (Fig. 4e).

We then evaluated the functionality of the differentiated insulin^+^ cells present in each sample by testing their capacity to secrete insulin following glucose stimulation (GSIS). Mature β-cells secrete insulin within minutes of glucose stimulation and will subsequently shut off the secretion to maintain glucose homeostasis. To determine whether our S7 cells contained cells capable of an accurate GSIS response, we performed a static incubation assay, including stimulating the S7 cells with high glucose (20 mM) alone, and supplemented with Exendin-4 (10 nM), a Glucagon-like protein 1 (GLP-1) receptor agonist. Finally, cell depolarization was tested with high glucose supplemented with 30 mM KCl (insulin secretion in Fig. 4f, c-peptide secretion in Fig. 4g). The static incubation assay revealed that our S7 cells are immature, as they show a decreased glucose threshold for insulin secretion^13^, and they release insulin in an uncontrolled manner with surprisingly lower insulin secretion at higher glucose concentrations (Fig. 4f). There were no observed HNF4A mutation-specific differences in the GSIS readout (Fig. 4f,g). These data match the insulin secretion observed from hESC-derived S7 cells reported previously^9^. We further confirmed these findings in assays where the cells were kept in low glucose overnight (1.67 mM) followed by extensive washings (glucose-free DPBS and Krebs buffers without glucose) before high glucose stimulation (20 mM). Moreover, proinsulin was not detected by ELISA confirming the absence of contaminating cells or cell debris in the media. These findings combined, rule out a technical error causing the low insulin and c-peptide release response observed. However, interestingly, insulin and c-peptide secretory response to KCl was increased compared to what have been reported for maturing β-like cells using the same protocol^9^.

To further analyse the S7 cells‘ maturity status we compared the proteome of S7 cells derived from the three MODY1 mutation carriers and their family control to the proteome of adult human islets. We chose islets as opposed to sorted human β-cells as the pancreatic islet is more similar to S7 cells regarding cell heterogeneity. Moreover, the FACS procedure is known to increase the mortality and stress of sorted β-cells hence potentially bringing unwanted global changes to their proteome distribution. The analysis revealed that, for some selected key pancreatic β-cell-markers, all the S7 cells, regardless of the MODY1 mutation status, exhibited similar abundance levels compared to human islets (HIs) (Fig. 4h, Fig. S2), while other human islets markers had a higher abundance in the human islets. One such example is Urocortin-3 (UCN3), a marker of functionally mature β-cells^19^, which showed increased protein level in human islets as compared to all S7 samples (MODY1 mutation carriers and the family control) (Fig. 4h, green background). Similarly, the abundance level of MAFB was lower in human islets, indicating that S7 cells, regardless of the mutation or diabetes status, are less mature than human islet cells (Fig. 4h, orange background). Moreover, despite the increased abundance of proteins from S6 to S7 involved in granulogenesis (Fig. S3), suggesting that the *in vitro*-generated insulin^+^ cells are capable of producing appropriately packed insulin granules and possess exocytosis machinery (Fig. 4a–e), our data also showed lower levels in S7 compared to islets of proconvertase 1 (PCSK1), a key actor in the insulin processing machinery. Compared to the reported expression level of PCSK1 in S7 cells^9^, we find the opposite regulation level as compared to human islets, suggesting an inefficient insulin processing machinery. This, in combination with decreased GSIS, points towards the immaturity status of the S7 cells in all MODY1 mutation carriers as well as in the family control.

Last, the proliferation marker KI67 was increased in the S7 cell population as compared to human islets, showing many of these cells retain their proliferative potential, further supporting the notion of an immature phenotype (Fig. 4h, red background).

### Comparative analysis of S7 cells and human islets reveals missing molecular landscape involved in β-cell fate

**Figure 5:**
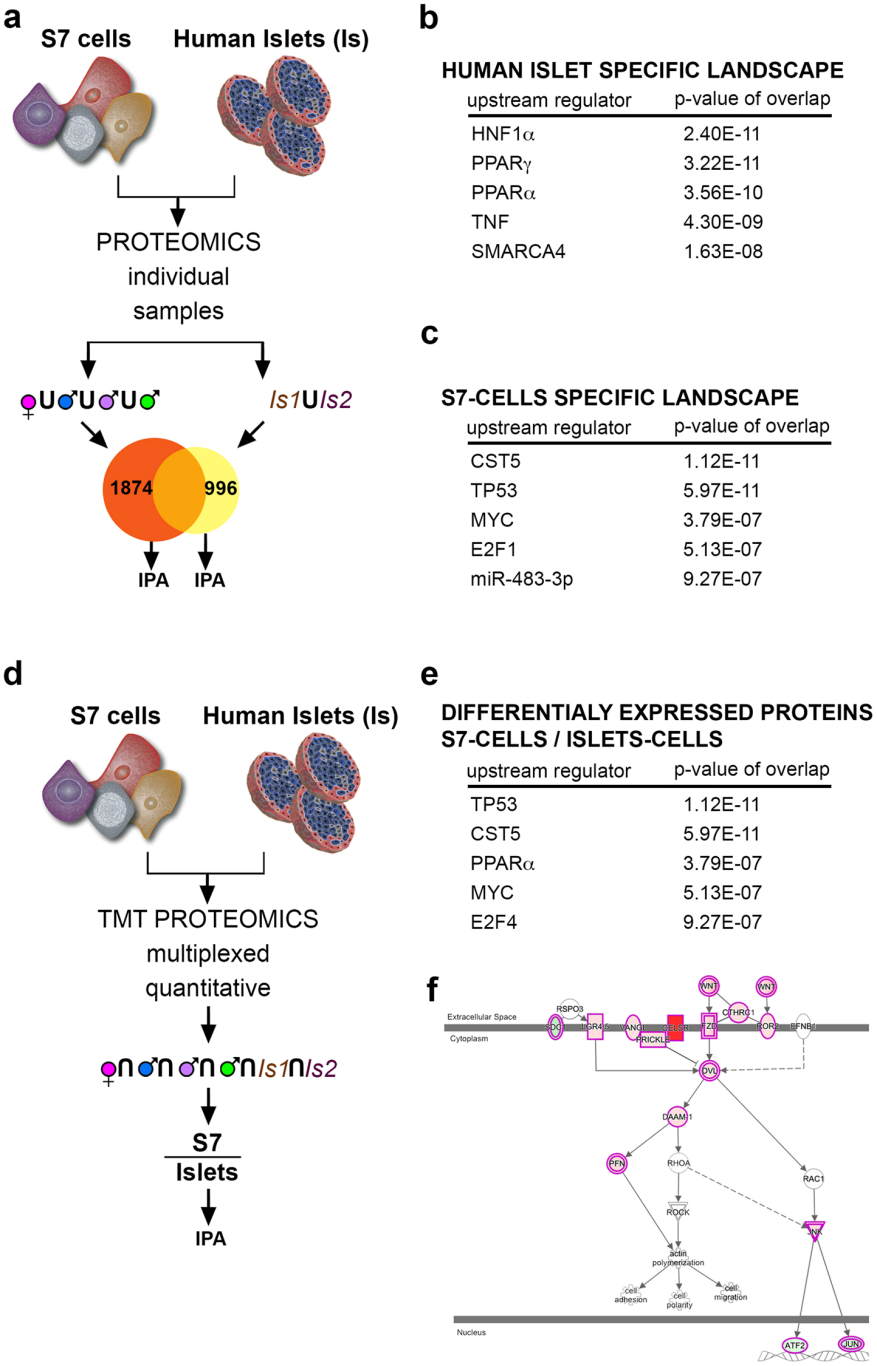
Comparative IPA analyses of the human islets and S7 cell population. (**a**) Experimental design depicting the IPA analysis of proteins that were identified exclusively in human islet-specific and S7-cells molecular, respectively. The data represents individual samples from the 10-plex. The proteome of *in vitro* generated S7 cells showed 1874 (yellow pie) uniquely identified proteins as compared to 996 (orange pie) uniquely identified in human islets. (**b**) The top 5 upstream regulators predicted from the human islet-specific molecular landscape. (**c**) The top 5 upstream regulators predicted from the S7-cell specific molecular landscape. (**d**) Experimental design depicting the analysis of the differentially expressed between S7 cell proteome compared and human islets. Note that samples were combined before they were run enabling relative quantification. (**e**) The top 5 upstream regulators predicted from the proteins differentially expressed between S7 cells and human islets. (**f**) PCP pathway components predicted as activated by IPA from the proteins differentially expressed between S7 cells and human islets.

Characterizing the inherent molecular differences between the artificially generated hiPSC-derived insulin^+^ cells and bona-fide β-cells is critical for improving the yield of the differentiation protocol and the quality of the end product. To address this critical point using proteomics on individual samples, we first focused on the proteins that were identified in only one of the main compared categories, i.e. in either the S7-cells (from the MODY1 quadro with three MODY1-mutation carriers and their family control) or in the human islets, but not in both (Fig. 5a). All proteins identified in at least one sample were selected for the corresponding condition grouping. We found 996 proteins uniquely identified in human islets and 1874 proteins uniquely identified in the S7 cells (MODY1 quadro S7P).

The Ingenuity Pathway Analysis (IPA) of the human islets specific proteome landscape predicted among the top 5 upstream regulators (i.e. regulators responsible for the observed protein landscape) important transcription factors of β-cell identity and function (Fig. 5b), with HNF1α (p-value of overlap 2.40E-11) and PPARγ (p-value of overlap 3.22E-11) as top regulators. A similar analysis of the S7-cells-specific proteome landscape revealed among the top 5 upstream regulators, transcription factors involved in cell cycle progression, such as TP53 (p-value of overlap 5.97E-11) and MYC (p-value of overlap 3.79E-07) (Fig. 5c). These results suggest that the S7 cells (from the MODY1 quadro) contain a significant fraction of proliferating cells. Moreover, as indicated by the human islets specific proteome landscape analysis, the S7 cells (from the MODY1 quadro) are also missing critical networks required for β-cell identity and function.

### S7 cells and human islet comparison reveals differentially regulated candidate molecular networks potentially involved in β-cell fate

We further performed pathway analysis on the proteins present in both S7 cells (from the MODY1 quadro) and human islets, but differentially expressed between the two conditions (FC ≥ 2, hiPSC-derived S7 as compared to human islets, Fig. 5d). The top 5 upstream regulators predicted by the analysis mostly consisted of genes involved in cell-cycle regulation, such as TP53 (predicted inhibition), MYC (predicted activation) and PPARA (Fig. 5e). Moreover, the top canonical pathway identified, the LXR/RXR pathway, which activation is known to block cell proliferation, was predicted as strongly inhibited (p-value 2.28E-13, z-score −4.621). Overall, these results are further confirming our previous observations of increased proliferation in the S7 population (see also KI67 in Fig. 4c). This biological behaviour is in clear contrast to the well-known low proliferation index characterizing the human islet, further confirming the immaturity of the S7 population.

The analysis also predicted the upregulation of key developmentally specific canonical pathways, such as Wnt/β-catenin signalling (p-value 2.7E-06, z-score 0.905) and PCP signalling (p-value 4.75E-05, z-score 2.558) (Fig. 5f). The strong canonical and non-canonical Wnt pathway activation in the S7 cells, as compared to the human islets is expected to affect the differentiation and maturation potential of insulin^+^ cells *in vitro*, its modulation being a potential target for improving the efficiency of the current differentiation protocols.

## Discussion

In this study, we demonstrate that, with the experimental set-up described, insulin^+^, MAFA^+^, NKX6.1^+^ S7 cells can be generated *in vitro* from hiPSCs regardless of *HNF4A* mutation carrier status or diabetes status. Furthermore, also regardless of *HNF4A* mutation carrier status or diabetes status, the S7 cells displayed the same GSIS kinetics, the same ultrastructural features and the same levels of proteomics-based protein markers of a mature β-cell, including INS, PDX1, NKX6.1, MAFA, GLUT2, UCN3 and others. These data are consistent with observations from hiPSC-derived β-cells from patients with type 1 diabetes^20^. Milleman *et al*., reports that using this model system, upon β-cell stress, the T1D-patient derived hiPSCs do not respond differently from non-diabetic hiPSC-derived β-cells. Since reprogramming into iPSC resets aspects of cellular and molecular aging^21^, progerin-induced aging of the *in vitro* generated iPSC-derived patient-specific cells could be one strategy to uncover disease specific traits^22^. Taken together, the above indicate that the trigger of the MODY1 phenotype/β-cell dysfunction acts downstream of β-cell differentiation, probably after the β-cells have been formed, suggesting mechanisms such as cell death upon stress or low clearance of the senescence markers. Nevertheless, as the *HNF4A* mutation described in this manuscript, p.Ile271fs, is not a null mutation, but rather a non-sense mutation generating a truncated HNF4A product, we cannot exclude an even stronger impact on β-cell differentiation in the case of the absence of a full allele also affecting differentiating events. Interestingly, there are no known human HNF4A null mutations known to date. Moreover, the available HNF4α general knockout murine model is embryonic lethal^23^. Taken together, these two observations suggest that the loss of a full *HNF4A* allele is probably embryonically lethal. However, if the loss of *HNF4A* is occurring in adult β-cells, by using a conditional knockout mouse, then the mice are viable but exhibit impairment of glucose-stimulated insulin secretion^24^.

Importantly, we find that the differentiated S7 cell population is characterized by a high cellular heterogeneity. Regardless of the *HNF4A* mutation and diabetes disease status, the S7 populations were characterized by a mix of different stages of progenitor cells, including mixed islet cell populations, as well as cells expressing different hormone combinations (including bihormonal cells). Mixed islet cell populations could also partly explain our observation that insulin secretion was seemingly reduced at high glucose concentrations compared to low glucose concentration since a subgroup of glucose-stimulated somatostatin secreting cells (given the observed somatostatin expression; Fig. 2c, and in accordance with a previously reported subpopulation of somatostatin+ S7 cells^9^) could potentially inhibit insulin secretion. Moreover, both the proteomic analysis and physiological tests showed that the generated insulin^+^ cells are immature and require further maturation to become fully functional β-like cells. Both the heterogeneity of the S7 fraction and the immaturity of the *in vitro* generated insulin-producing cells are not surprising and have been previously reported in most widely used β-cells differentiation protocols. These problems may, however, affect safety issues such as a potential carciogenesis risk conferred from the multiprogenitor potential in clinical therapeutic studies aimed at transplanting S7 cells as β-cell therapy. Consequently, further efforts are required for refining the current *in vitro* strategies towards generating β-like cells with physiological GSIS kinetics, similar to the mature bona-fide β-cells.

Surprisingly, transplantation of *in vitro* differentiating cells into immunodeficient mice promotes further their *in vivo* differentiation and maturation, and has proven to be successful for curing induced diabetes in mice^25^. This observation suggests the presence of *in vivo* maturation factor(s) missing from the current β-cell differentiation protocols. To reveal these missing molecular components, we performed a global proteome analysis of immature (S6) and maturing (S7) cells derived from MODY1 mutation carriers and a family control and compared to the human islet proteome. Many studies have been performed to generate reference maps of the human pancreatic islet proteome^26^. Previously, the most comprehensive human pancreatic islet study identified 3,365 proteins^27^. By mapping the global human islet cell proteome, we quantified 8,800 proteins. To our knowledge this is the largest number of identified proteins reported for human pancreatic islets, and these data have provided us with unparalleled insights into the specific proteome landscapes of pancreatic islets. There is a potential bias in our analyses introduced by heterogeneity of varying proportions in the cellular mix of the clones vs islets. In addition bias may be introduced by islets have natural variability in the proportions of different subpopulations of cells and by contamination of human islet preparations with acinar and ductal structures as well as bias introduced by stress associated with islet isolation^9^.

**Figure 6:**
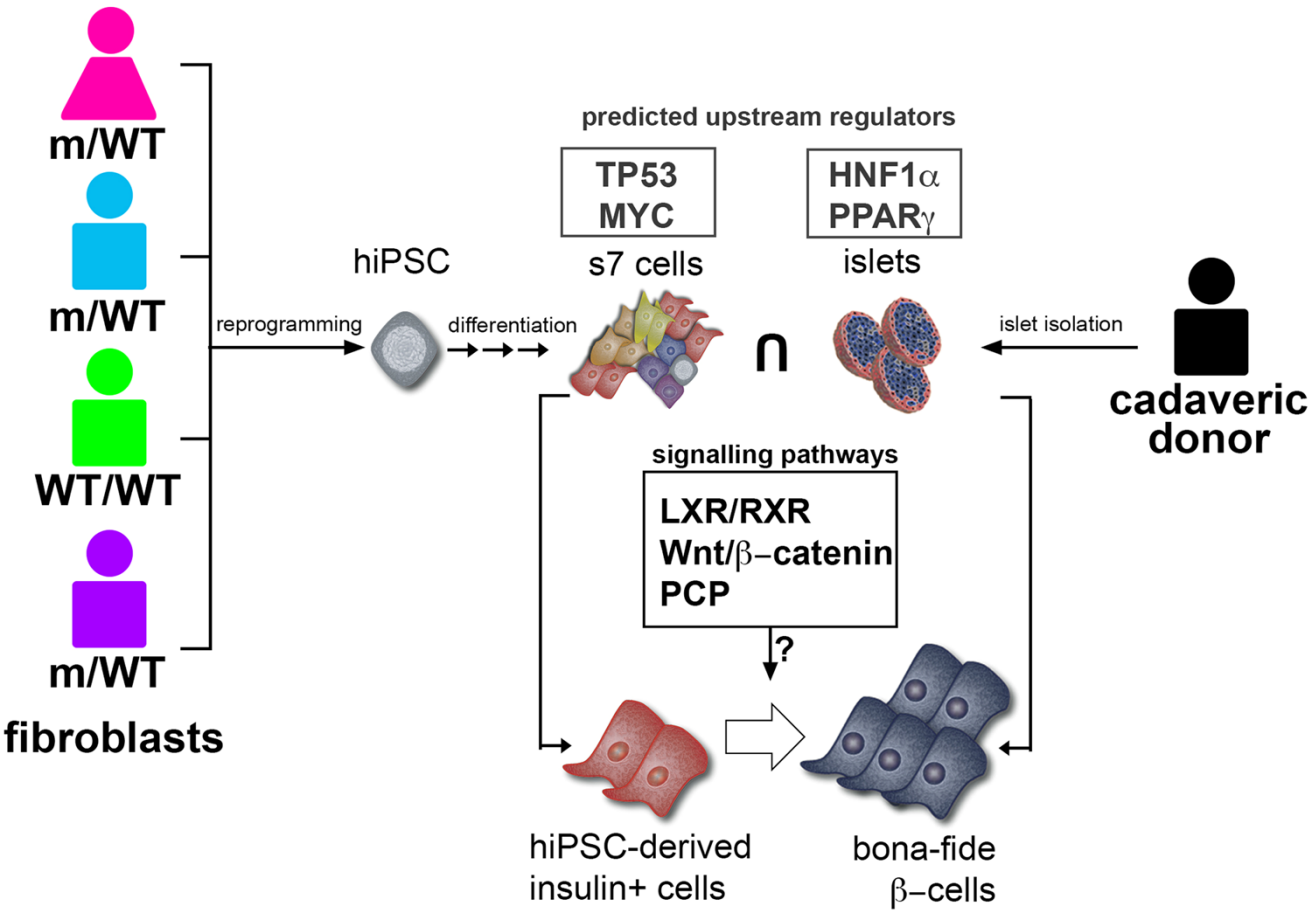
Comparison of hiPSC-derived insulin-producing cells and human islets suggesting missing molecular landscape involved in β-cell fate. For the differentiation of patient-specific S7 insulin-producing cells, we reprogrammed skin fibroblasts from four donors from the same family (n = 4), including a parental diabetic mutation carrier, and three offspring; one diabetic mutation carrier, one non-diabetic mutation carrier, and non-diabetic non-mutation carrier (family control). We found that positive *HNF4A* mutation status does not prevent the formation of insulin positive S7 cells. However, in agreement with previous reports, these S7 cells are immature and require further maturation to become fully functional β-like cells. Specifically addressing this absent maturation of S7 cells, we show that the careful assessment of the proteome of these differentiating cells compared to the human islet proteome can be exploited to detect factors that are deficient or overexpressed, factors that might enable the *in vitro* maturation towards fully functional β-cells. The human islets specific proteome landscape predicted HNF1α and PPARγ as upstream regulators important for β-cell identity and function. A similar analysis of the S7-cells-specific proteome landscape predicted TP53 and MYC as upstream regulators transcription factors involved in cell cycle progression. These results suggest that the S7 cells contain a significant fraction of proliferating cells. Moreover, as indicated by the human islets specific proteome landscape analysis, the S7 cells are also missing critical networks required for β-cell identity and function.

The comparative analysis of S7 cells from the MODY1 quadro to human islets revealed a missing molecular landscape involved in β-cell fate (Fig. 6). Interestingly, we found differences in the proteomic landscape under the presumptive regulation of *HNF1A* (particular to islets). Moreover, we also identified several signalling pathways with known β-cell developmental and differentiation potential in the human islets samples, which exhibited a differentially regulated pattern in the S7-cells fraction, regardless of the MODY1 mutation status. One such example is the predicted upregulation of the canonical and non-canonical Wnt pathways in the S7 cells of all MODY1 quadro samples as compared to the human islets. The modulation of these pathways^28^ is expected to affect the differentiation and maturation potential of insulin^+^ cells *in vitro*, thereby uncovering potential targets for improving the efficiency of generating β-like cells *in vitro*.

## Materials and Methods

### Cell source

Human islets (n = 3) were isolated as described previously^29^ from pancreata obtained from two female and one male brain-dead deceased donors after informed consent from relatives for organ donation and for use in research at the islet isolation facility of Oslo University Hospital, Oslo, Norway. In this study, we present data from two human islets samples, one of the samples were excluded due to technical issues. All experiments with human islets was approved by the regional committee for medical and health research ethics central in Norway (2011/782).

The human induced pluripotent stem cells iPSCs used in this study were episomal reprogrammed from four family members with MODY1 diabetes, including a healthy family control. The iPSCs cell lines were characterized and confirmed to have normal karyotype, and to be mycoplasma free using a MycoAlert Mycoplasm Detection Kit (Lonza, LT07-418). All hiPS cell lines were subjected to Stage-specific embryonic antigen 4 (SSEA4) enrichment (SSEA4 microbeads, MACS Miltenyi Biotec) before proceeding to differentiation.

All samples used for differentiation and further analyses throughout the manuscript were generated from a MODY1 quadro, i.e. three *HNF4A* mutation carriers (two with overt diabetes and one non-diabetic mutation carrier) and their family control (healthy male sibling without mutation).

All experiments with hiPSCs were approved by the Regional Committee of Medical and Health Research Ethics (REK 2010/2295), and all methods were performed in accordance with the Helsinki Declaration.

### In vitro differentiation protocol

Four iPSC lines (three MODY1 mutation carriers and their family control) were differentiated according to the protocol^9^ on Matrigel-coated plates for the duration of the whole differentiation protocol, without transfer to an air-liquid interface. The cells were grown in stage 6 media and stage 7 media for 7 days, respectively. iPS-derived cells were harvested for downstream immunofluorescence staining at each of the seven stages of differentiation. We harvested iPS-derived cells for proteomics analysis at Stage 6 (n = 2) and 7 (n = 4) corresponding to immature and maturing beta cells, respectively. Stage 7 cells were further subjected to Glucose Stimulated Insulin Secretion (GSIS) and Transmission Electron Microscopy (TEM).

### Static incubation glucose-stimulated insulin secretion Stage 7 cells

Stage 7 cells were rinsed twice with Krebs buffer (129 mM NaCl, 5 mM NaHCO_3_, 4.8 mM KCl, 2.5 mM CaCl_2_, 1.2 mM MgSO_4_, 1 mM Na_2_HPO_4_, 1.2 mM KH_2_PO_4_, 10 mM HEPES, 0.1% BSA) in deionized water and then sterile filtered. The cells were pre-incubated in Krebs buffer for 30 min. Followed by incubation in Krebs buffer spiked with 1.67 mM glucose (Krebs buffer low glucose) for 40 min. Media was collected after 20 min and 40 min, centrifuged to remove unwanted cells and supernatant was transferred to a new vial. After each incubation, we added the same amount of fresh media to the cells. After low glucose incubation, the cells were incubated in Krebs buffer spiked with 20 mM glucose (Krebs buffer high glucose) for 60 min, and media was collected every 10 min. Followed by incubation in Krebs buffer spiked with 20 mM glucose and 10 nM Exendin-4 for 20 min, with media collection after 10 min and 20 min. As a last stage of stimulation of insulin secretion, cells were incubated in Krebs buffer spiked with 20 mM glucose and 30 mM KCl for 20 min, we collected media after 10 min and 20 min. All supernatants were frozen at −80 °C, and Ultrasensitive C-peptide levels (#10-1141-01;Mercodia), Insulin (#10-113-01; Mercodia) were measured with ELISA. Proinsulin (#10-1118-01; Mercodia) was measured as a negative control, to check that cells were removed from the media.

### Fixation and embedding of cells for Transmission electron microscopy (TEM)

Cells were fixed in 2% glutaraldehyde in 0.1 M sodium cacodulate buffer for two hours, followed by washing with 0.1 M sodium cacodulate buffer (3 × 15 min). Post fixation was done in 1% OsO4 60 min, followed by washing with 0,1 M sodium cacodulate buffer (2 × 10 min). Dehydration in a gradient of 30%, 50%, and 70% ethanol for 15–20 min, and 96% followed by 100% ethanol for 20 min. Cells were embedded in 50:50 ethanol/resin and a second round in 100% Resin. Cells were allowed to harden at 60 °C for 48 hours.

### Immunofluorescence

Cells were cultured on glass coverslips and fixed in 2% PFA for 15 min. The immunofluorescence protocol was performed conform indications provided by the supplier. The antibodies used are given in Tables 1 and 2.

**Table 1:** Primary antibodies.

Primary antibody	Dilution	Company	Catalog #
**rabbit anti-human FOXA2**	1/200	Abcam	ab23630
**goat anti-human Sox17**	1/40	R&D Systems	AF1924
**mouse anti-human HNF-4 α**	1/1000	Abcam	ab41898
**goat anti-human HNF-1 β**	1/20	R&D Systems	AF3330
**Rabbit anti-human MAFA**	1/200	Abcam	ab98859
**rabbit anti-human NKX6.1**	1/100	Novus	NBP1-82553
**guinea-pig anti-porcine insulin**	1/400	Dako	A056401-2
**mouse anti-porcine glucagon**	1/1000	Sigma	G2654

**Table 2:** Secondary antibodies.

Secondary antibody	Dilution	Company
**donkey anti-rabbit A488**	1/500	Molecular Probes
**goat anti-guinea-pig A488**	1/500	Molecular Probes
**donkey anti-rabbit A546**	1/500	Molecular Probes
**donkey anti-mouse A594**	1/500	Molecular Probes
**donkey anti-goat A647**	1/500	Molecular Probes
**goat anti-guinea-pig A647**	1/500	Molecular Probes

The nuclei were stained with DAPI (D1306, Molecular Probes). The samples mounted in Prolong Diamond Antifade Mountant Media (P36970, Life technologies) and were analyzed with Leica TCS SP2 AOBS or Leica TCS SP5 STED CW confocal microscopes. No specific feature of the original data was obscured, eliminated or misrepresented.

### Global proteomics analysis

#### Cell lysis and protein digestion

Cell cultures were harvested with Gentle Cell Dissociation Reagent, followed by centrifugation. 200 handpicked equally sized islets were collected in column, washed 1XPBS and islet pellet was stored at −70 °C until lysis. Cells (S6, S7 and islets) were lysed in buffer containing 4% SDS, and boiled at 95 °C for 7 min on a shaker, and sonicated (three rounds a 30 sec, 30% power. The protein concentration was determined using a BCA protein assay kit. Dry aliquots containing an estimated amount of 100 µg of proteins were further processed using Filter-Aided Sample Preparation^30^.

#### Tandem Mass Tag (TMT) 10-plex labelling

Our approach using TMT 10-plex labelling allowed for 10 samples to be analysed simultaneously; human islets (n = 3), S6 cells (n = 2), S7 cells (n = 4), and we also included one sample containing a mix of equal amounts from a different MODY family (data not shown). TMT reagents were re-suspended in ACN. Desalted peptides were re-suspended in 100 μL of 200 mM HEPES pH 8.5, 30 μL of ACN, and 10 μL of the TMT reagents were added to the respective peptide samples, gently vortexed, and incubated for 1.5 h at RT. To prevent unwanted labelling, the reaction was quenched by adding 10 μL of 5% hydroxylamine and incubated for 15 min at RT. Equal amounts of the TMT-labelled samples were combined and concentrated to near dryness, followed by desalting via C18 solid phase extraction. TMT 10-plex labelling allows for relative quantification of peptides across ten samples. The intensity (abundance) of a labelled peptide in one sample is quantitatively compared to the intensity of the same labelled peptide in ten samples in parallel.

#### Off-line basic pH reversed phase fractionation

The combined labelled peptide samples were pre-fractionated by basic pH reversed phase HPLC as described previously^31^, using an Agilent (P/N 770995-902) 300Extend-C18, 5 μm, 250 mm × 4.6 mm id column, connected to an Agilent Technology off-line LC-system. Solvent A was 5% ACN, 10 mM NH_4_HCO_3_ pH8, and solvent B was 90% ACN, NH_4_HCO_3_ pH 8. The samples were re-suspended in 500 μL solvent A and loaded onto the column. Column flow was set to 0.8 mL/min and the gradient length was 70 min, as follows: from 0–35 min solvent 50% A/ 50% B, and from 35–50 min 100% B, and from 50–70 min 100% A. The labelled peptides were fractionated into 96 fractions, and further combined into a total of 12 fractions. Each fraction was acidified with 1% formic acid, concentrated by vacuum centrifugation to near dryness, and desalted by StageTip. Each fraction was dissolved in 5% ACN/ 5% formic acid for LC-MS/MS analysis.

#### LC-MS3 analysis

From each of the 12 fractions, ~5 μg was dissolved in 1% aqueous formic acid (FA) prior to LC-MS/MS analysis on an Orbitrap Fusion mass spectrometer (Thermo Fisher Scientific, San Jose, CA) coupled to a Proxeon EASY-nLC 1000 liquid chromatography (LC) pump (Thermo Fisher Scientific). Peptides were fractionated on a 75-μm inner diameter microcapillary column packed with ~0.5 cm of Magic C4 resin (5 μm, 100 Å, Michrom Bioresources) followed by ~35 cm of GP-18 resin (1.8 μm, 200 Å, Sepax, Newark, DE). For each analysis, we loaded ~1 μg onto the column.

Peptides were separated using a 3 hr gradient of 6 to 26% acetonitrile in 0.125% formic acid at a flow rate of ~350 nL/min. Each analysis used the multi-notch MS3-based TMT method^32^ on an Orbitrap Fusion mass spectrometer, which has been shown to reduce ion interference compared to MS2 quantification^31^. The scan sequence began with an MS1 spectrum (Orbitrap analysis; resolution 120,000; mass range 400–1400 m/z; automatic gain control (AGC) target 2 × 10^5^; maximum injection time 100 ms). Precursors for MS2/MS3 analysis were selected using a TopSpeed of 2 sec. MS2 analysis consisted of collision-induced dissociation (quadrupole ion trap analysis; AGC 4 × 10^3^; normalized collision energy (NCE) 35; maximum injection time 150 ms). Following acquisition of each MS2 spectrum, we collected an MS3 spectrum using our recently described method^32^ in which multiple MS2 fragment ions were captured in the MS3 precursor population using isolation waveforms with multiple frequency notches. MS3 precursors were fragmented by high-energy collision-induced dissociation (HCD) and analysed using the Orbitrap (NCE 55; AGC 5 × 10^4^; maximum injection time 150 ms, resolution was 60,000 at 400 Th).

### Data analysis

Mass spectra were processed using a Sequest-based in-house software pipeline^33^, and spectra were converted to mzXML using a modified version of ReAdW.exe. Database searching included all entries from the human uniprot database (March 11, 2014). This database was concatenated with one composed of all protein sequences in the reversed order. Searches were performed using a 50 ppm precursor ion tolerance for total protein level analysis. The product ion tolerance was set to 0.9 Da. These wide mass tolerance windows were chosen to maximize sensitivity in conjunction with Sequest searches and linear discriminant analysis^33, 34^. TMT tags on lysine residues and peptide N termini (+229.163 Da) and carbamidomethylation of cysteine residues (+57.021 Da) were set as static modifications, while oxidation of methionine residues (+15.995 Da) was set as a variable modification.

Peptide-spectrum matches (PSMs) were adjusted to a 1% false discovery rate (FDR)^35, 36^. PSM filtering was performed using a linear discriminant analysis, as described previously^33^, while considering the following parameters: XCorr, ΔCn, missed cleavages, peptide length, charge state, and precursor mass accuracy. For TMT-based reporter ion quantitation, we extracted the summed signal-to-noise (S/N) ratio for each TMT channel and found the closest matching centroid to the expected mass of the TMT reporter ion.

The search space for each reporter ion was limited to a range of 0.003 Th to prevent overlap between the isobaric reporter ions. For protein-level comparisons, PSMs were identified, quantified, and collapsed to a 1% peptide false discovery rate (FDR) and then collapsed further to a final protein-level FDR of 1%. Moreover, protein assembly was guided by principles of parsimony to produce the smallest set of proteins necessary to account for all observed peptides.

Proteins were quantified by summing reporter ion counts across all matching PSMs using in-house software, as described previously^33^. PSMs with poor quality, MS3 spectra with more than eight TMT reporter ion channels missing, MS3 spectra with TMT reporter summed signal-to-noise ratio that is less than 100, or no MS3 spectra were excluded from quantitation^37^. Protein quantitation values were exported for further analysis in Microsoft Excel or SAS JMP. Each reporter ion channel was summed across all quantified proteins and normalized assuming equal protein loading of all 10 samples.

For the all the global proteome comparisons referred to throughout the manuscript we used samples from MODY1 mutation carriers and their family control and compared with human islet samples.

The pathway analyses were generated through the use of QIAGEN’s Ingenuity Pathway Analysis (IPA®, QIAGEN Redwood City, www.qiagen.com/ingenuity). Briefly, the analyses were performed with the following settings: Expression Value Type (Exp Log Ration), Reference set (Ingenuity Knowledge Base + Endogenous chemicals), Relationships to consider (Direct and Indirect Relationships), Interaction networks (70 molecules/network; 25 networks/analysis), Data Source (all), Confidence (Experimentally Observed), Species (Human, Mouse, Rat), Tissue & Cell Lines (all), Mutations (all).

We applied non-parametric tests to assess for significant differences in protein abundance in the clones or islets. We chose a significance level of 5% and used GraphPad Prism (GraphPad software, version 7, La Jolla, CA).

## Electronic supplementary material

Supplementary Information
