# Supplementary material for: Probing the missing mature β-cell proteomic landscape in differentiating patient iPSC-derived cells
Source: Sci Rep. 2017 Jul 6;7:4780. doi: 10.1038/s41598-017-04979-w (PMC5500592; doi:10.1038/s41598-017-04979-w)
Supplement: Supplementary file 1 — Supplementary Information [file 41598_2017_4979_MOESM1_ESM.pdf]

Supplementary Information

Probing the missing mature β-cell proteomic landscape in differentiating patient iPSC-derived cells

Heidrun Vethe[1], Yngvild Bjørlykke[4], Luiza M. Ghila[1], Joao A. Paulo[2], Hanne Scholz[3], Steven P. Gygi[2], Simona Chera[1] & Helge Ræder[1, 4]

[1]KG Jebsen Center for Diabetes Research, Department of Clinical Science, University of Bergen, Bergen, Norway

[2]Department of Cell Biology, Harvard Medical School, Boston, MA, USA

[3]Department of Transplant Medicine, Oslo University Hospital, Oslo, Norway

[4]Department of Pediatrics, Haukeland University Hospital, Bergen, Norway

Correspondence:

Helge.Rader@uib.no
Tel: +47 55975263
Fax: +47 55975289

<u>Supplementary data:</u>

Figure S1. Differentiation of patient-specific iPSC into insulin-producing cells. a) Schematic representation of cell stages during differentiation of patient-specific iPSCs from four family members from a MODY1 family **b)** Co-expression of FoxA2 (green) and Sox17 (red) in S1 cells; HNF4alpha (green) and HNF1beta (red) in S3 cells; MAFA (green) and insulin (red) in S5 cells and NKX6.1 (green) and insulin (red) in S7 cells. Scale 50 µm.

Figure S2. Comparative analysis of S7 cells vs Human Islets. Fold difference (log2) between S7 and Human islets

Figure S3. β-cell markers grouped after function. The charts show relative abundance (log2) of insulin between S6, S7 and human islets, along with proteins involved maturation, proliferation, glucose sensing and insulin biosynthesis, beta cell transcription factors, regulation of GSIS, and granulogenesis.

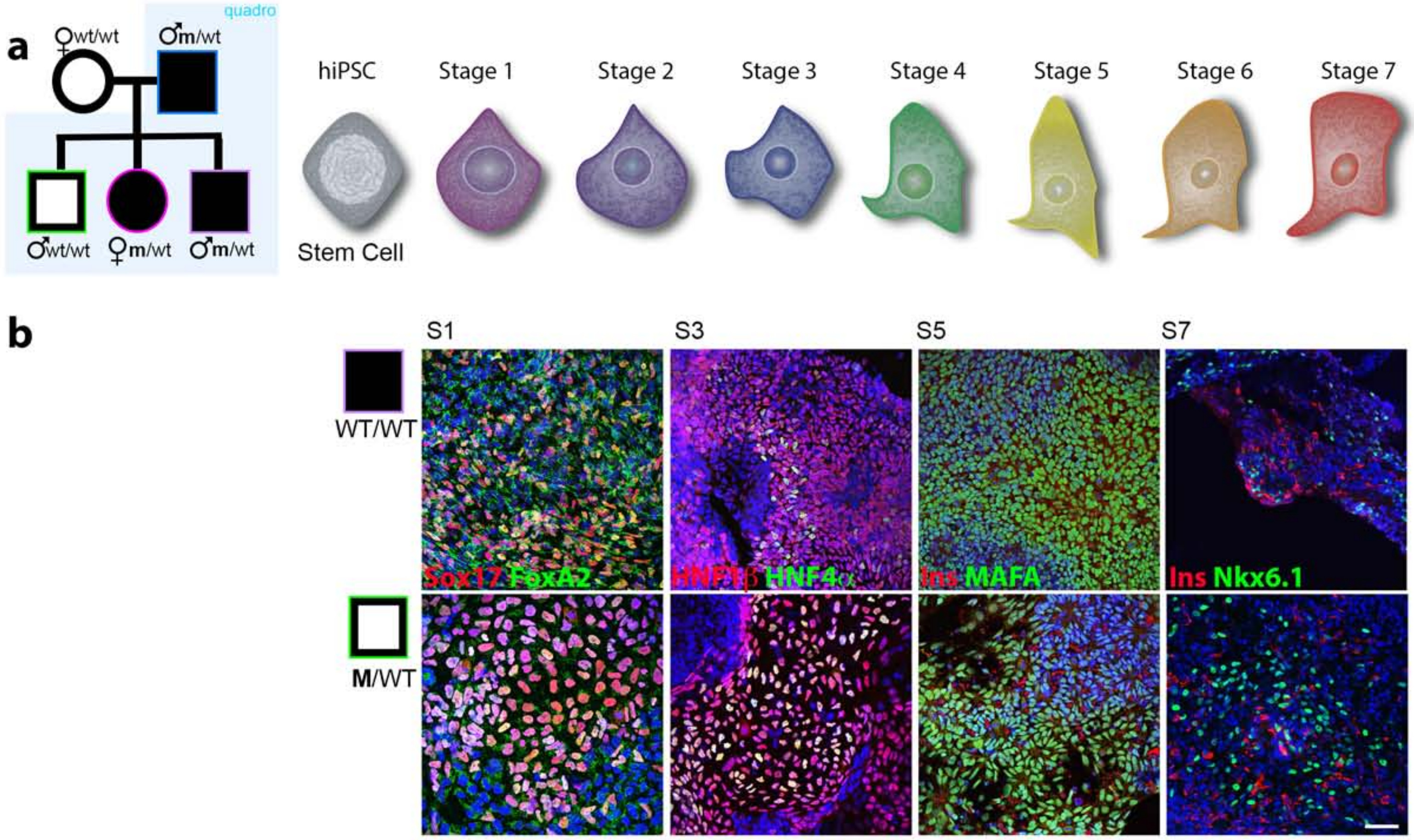

Vethe et al., supplemental figure 1

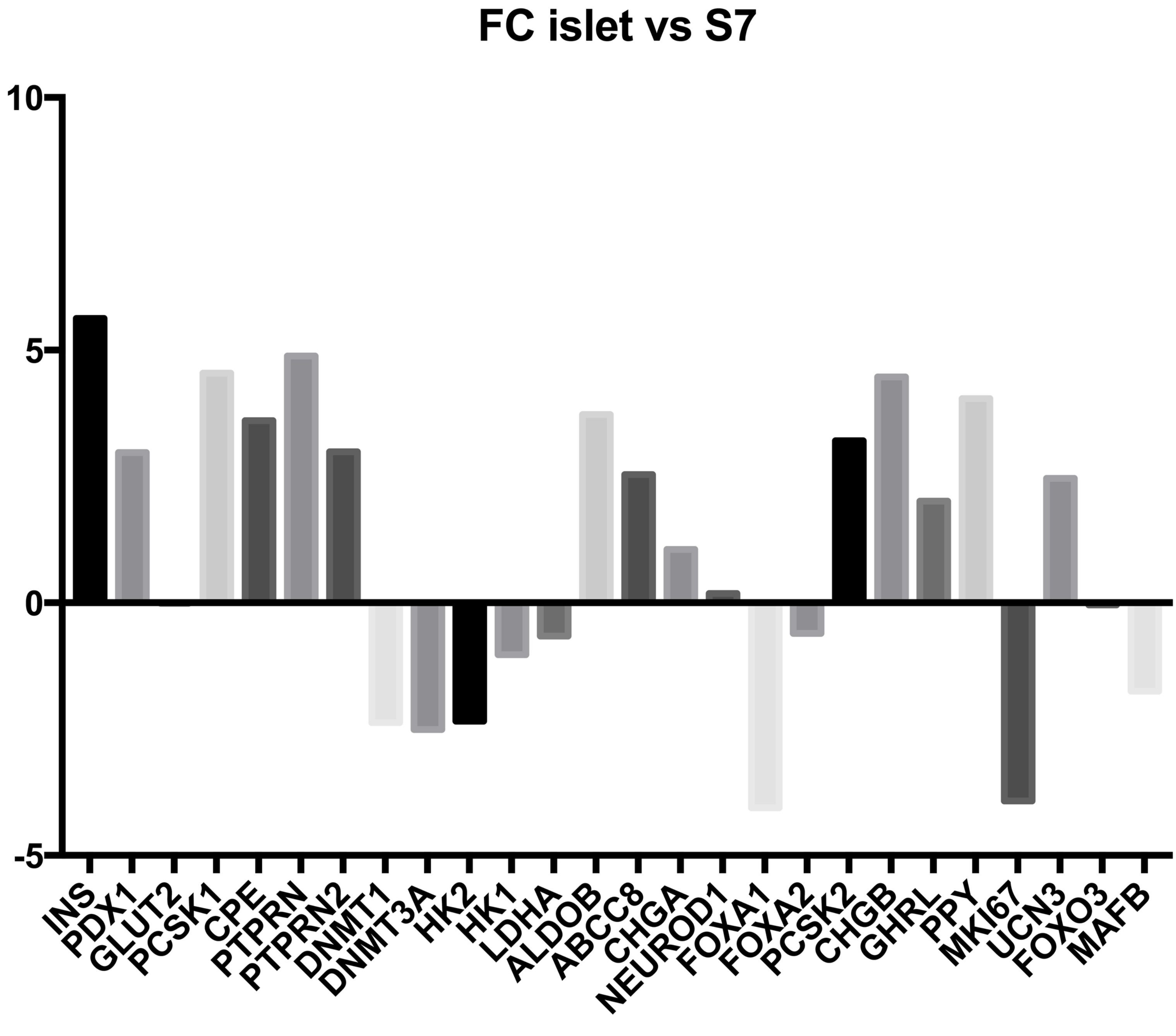

FC islet vs S7
10
5
0
-5
INS
PDX1
GLUT2
PCSK1
CPE
PTPRN
PTPRN2
DNMT1
DNMT3A
HK2
HK1
LDHA
ALDOB
ABCC8
CHGA
NEUROD1
FOXA1
FOXA2
PCSK2
CHGB
GHRL
PPY
MKI67
UCN3
FOXO3
MAFB

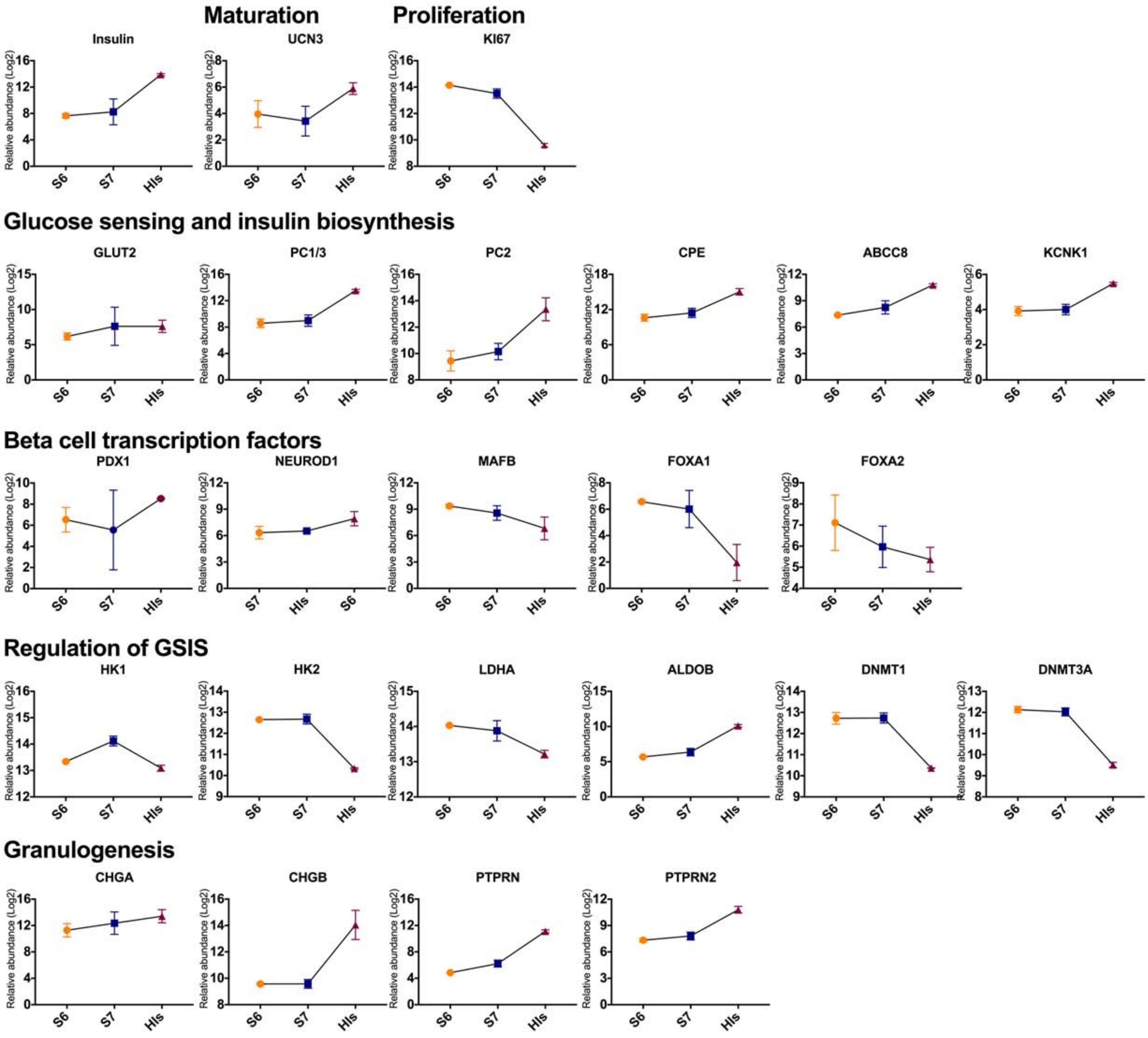
Maturation
Proliferation
Insulin
UCN3
KI67
Glucose sensing and insulin biosynthesis
GLUT2
PC1/3
PC2
CPE
ABCC8
KCNK1
Beta cell transcription factors
PDX1
NEUROD1
MAFB
FOXA1
FOXA2
Regulation of GSIS
HK1
HK2
LDHA
ALDOB
DNMT1
DNMT3A
Granulogenesis
CHGA
CHGB
PTPRN
PTPRN2
Relative abundance (Log2)
S6
S7
HIs
